# The Preventable Risk Integrated ModEl and Its Use to Estimate the Health Impact of Public Health Policy Scenarios

**DOI:** 10.1155/2014/748750

**Published:** 2014-09-25

**Authors:** Peter Scarborough, Richard A. Harrington, Anja Mizdrak, Lijuan Marissa Zhou, Aiden Doherty

**Affiliations:** ^1^British Heart Foundation Centre on Population Approaches to Non-Communicable Disease Prevention, Nuffield Department of Population Health, University of Oxford, Oxford OX3 7LF, UK; ^2^DCU School of Computing, Dublin City University, Dublin, Ireland

## Abstract

Noncommunicable disease (NCD) scenario models are an essential part of the public health toolkit, allowing for an estimate of the health impact of population-level interventions that are not amenable to assessment by standard epidemiological study designs (e.g., health-related food taxes and physical infrastructure projects) and extrapolating results from small samples to the whole population. The PRIME (Preventable Risk Integrated ModEl) is an openly available NCD scenario model that estimates the effect of population-level changes in diet, physical activity, and alcohol and tobacco consumption on NCD mortality. The structure and methods employed in the PRIME are described here in detail, including the development of open source code that will support a PRIME web application to be launched in 2015. This paper reviews scenario results from eleven papers that have used the PRIME, including estimates of the impact of achieving government recommendations for healthy diets, health-related food taxes and subsidies, and low-carbon diets. Future challenges for NCD scenario modelling, including the need for more comparisons between models and the improvement of future prediction of NCD rates, are also discussed.

## 1. Introduction

Noncommunicable diseases (NCDs) are the largest cause of ill health and mortality, responsible for 54% of disability adjusted life years lost in 2010 globally and over 80% in the developed world [1]. In Europe, cardiovascular diseases, cancer, and respiratory diseases account for 76% of all deaths [2]. Much is known about the epidemiology of NCDs thanks to pioneering work investigating causes of disease including ecological correlation studies [3], case control studies [4, 5], prospective cohort studies [5–8], and randomised controlled trials [9, 10]. This broad body of evidence has allowed us to understand how an individual's lifestyle behaviour affects their risk for NCD, which in turn has allowed for the development of diagnostic tools such as the Framingham Risk Score [11] and the QRISK score [12]. Similar techniques that are used to combine risk from different behaviours in individuals can also be used to assess the total risk of disease attributable to behavioural risk factors in a population, and these estimates can then be used to estimate the change in NCD risk within a population under counterfactual scenarios where the distribution of behavioural risk factors within the population is changed.

This has allowed for the development of a number of NCD scenario models such as the PRIME [13], the CHD Policy Model [14], and the ACE-Obesity model [15] and similar work considering improvements in physical inactivity [16], IMPACT [17], the RIVM DYNAMO model [18], and the UK Health Forum microsimulation model [19]. The purpose of these models is to estimate the impact on NCD morbidity and mortality of population-wide interventions aimed at improving health-related behaviour. The results of scenario modelling can be useful for a number of reasons. They can help with priority setting by comparing the health impact of changes in different behavioural risk factors, thereby identifying which risk factors could potentially deliver large health gains. For example, models can estimate the population health impact of achieving different dietary recommendations on the basis of the amount of disease within the population and current dietary patterns within the population. Such analyses can indicate whether achieving the population dietary goal for fruit and vegetables or the population dietary goal for salt would have a bigger impact on NCD mortality, for example, [20]. Scenario modelling can estimate the effectiveness of public health interventions when randomised trials are either impractical or unethical, for example, in the case of proposed taxes on sugar-sweetened beverages, where a randomised trial would involve randomising people, shops, or areas to receive increases in food prices. Scenario modelling can also be useful in identifying and mapping uncertainty in order to inform future research agenda. For example, a model of the impact of minimum pricing on alcohol consumption [21] and subsequent health outcomes involves a number of stages and assumptions: modelling the effect of changes in price on alcohol purchases (via price elasticities), modelling the effect of changes in purchases in changes in consumption, estimating the baseline distribution of alcohol consumption in the population, and modelling the effect of changes in alcohol consumption on the incidence of alcohol-related disease. Suitable analytical techniques, including sensitivity analyses and the production of tornado plots, can demonstrate how uncertainty in each of these areas affects the confidence in the final scenario results, thereby indicating where more research is needed in order to build a stronger evidence base.

A well-designed and transparent NCD model can act as a synthesis of current epidemiological knowledge, providing answers to “given what we already know, what if…” questions and scenarios. In order to have faith in the results of models, the structure must be transparently described and open to scrutiny, but due to their internal complexity NCD scenario models are often difficult to describe within the restrictions of health journals and difficult to share with peer reviewers. The Preventable Risk Integrated ModEl (PRIME) is described in this paper in detail and will be freely available for use as a web application from 2015 and available on request from the authors in advance. The source code supporting the PRIME web application will also be made freely available for download.

The structure of this paper is as follows. First, the structure of the PRIME and its potential use as an epidemiological tool are described. Then the detailed methods supporting the PRIME are described followed by the development of the source code for the PRIME web application. The following section provides a review of previous studies that have used the PRIME, or forerunners of the model. Then future development of the PRIME is described, and the paper concludes with a discussion of challenges in the field of NCD scenario modelling.

## 2. The Structure of the PRIME 

**Figure 1:**
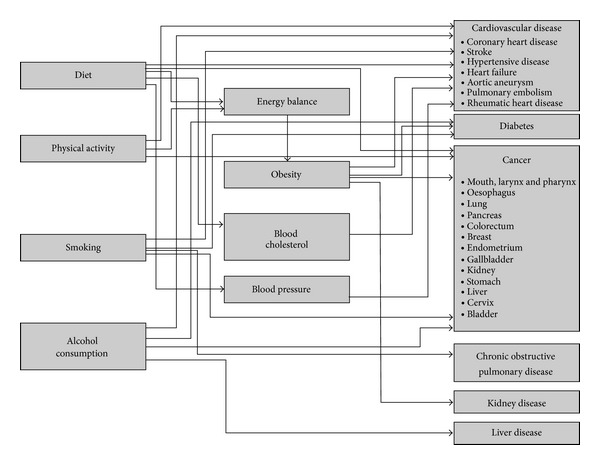
Schematic diagram of the PRIME.

The PRIME is a NCD scenario model that links behavioural risk factors with NCD mortality either directly or mediated by body mass index (BMI), blood cholesterol, or blood pressure. With a few exceptions (described under “Statistical Methods Used by the PRIME”), each of the links in the PRIME is parameterised by published meta-analyses of epidemiological studies: prospective cohort studies for links that terminate with NCD mortality and randomised controlled trials for links that terminate with either blood cholesterol or blood pressure. The model contains twelve behavioural risk factors covering the domains of diet, physical inactivity, alcohol consumption, and tobacco consumption. There are twenty-four health outcomes included in the PRIME, primarily cardiovascular diseases and cancers, but also kidney disease, liver disease, and chronic obstructive pulmonary disease. The structure for the PRIME is shown in Figure 1.

The PRIME is designed to estimate the impact on population NCD mortality of changes in the distribution of behavioural risk factors within that population. The user of the PRIME must enter age and sex specific estimates of the annual number of deaths from each NCD included in the model for the population of choice, as well as age and sex specific estimates of the number of people living in the population. They must then describe the baseline distribution of behavioural risk factors for their population, usually using microdata from a national health survey to estimate the mean and standard deviation of the variables of interest. The PRIME allows for age and sex specific estimates of behavioural risk factors refined up to five-year age bands—in practice most national health surveys are not adequately powered to estimate distributions at that level of refinement, so the user can choose a broader age range for the baseline input. Once the PRIME is suitably parameterised with this baseline data, the user can then enter any counterfactual scenario that they chose. A counterfactual scenario consists of a change in the distribution of one or more age and sex specific behavioural risk factor distributions. These counterfactual scenarios could be data driven, for example, applying the results of a change in food consumption due to a tax scenario estimated using price elasticity data [22], or theory-based, for example, estimating the impact of achieving a theoretical minimum risk distribution [23, 24]. Once the counterfactual scenario has been entered, the PRIME estimates the change in the annual number of NCD deaths between the baseline and counterfactual scenarios. Uncertainty intervals are calculated based on 5,000 iterations of a Monte Carlo analysis which allows the epidemiological parameters to randomly vary according to the distribution described in the literature. A complete list of parameters used in the PRIME is provided in Table 1.

The structure of the PRIME was designed in order to minimise the risk of double counting of effect size, by including epidemiologic parameters that have been appropriately adjusted for other behavioural risk factors. For example, the link between physical activity and health outcomes (described in more detail below) operates via two pathways: by influencing body weight and by the direct link between physical activity and health outcomes. The parameters used for the direct link are taken from meta-analyses where the relative risk included in the meta-analysis had been adjusted for body weight. In most cases, the association between the behavioural risk factor and the health outcome is modelled directly without adjustment for any mediating variables. For example, the link between alcohol consumption and coronary heart disease (CHD) is modelled directly without adjustment for the effect of alcohol on blood pressure—this implies that including a second link between alcohol consumption and blood pressure would introduce double counting since changing alcohol consumption levels would affect CHD rates via two pathways. Where mediating variables are modelled in the pathway (e.g., salt consumption to blood pressure to CHD), the corresponding direct association between the risk factor and the disease is either not modelled or is modelled using results adjusted for the mediating variable. Despite these attempts, it is likely that double counting remains in the model, as statistical adjustment in observational studies is unlikely to account for all potential confounding factors particularly when based on measurements taken at one time point only [41].

We used a set of inclusion criteria to decide whether or not to include a risk factor-disease relationship within the PRIME. These criteria were as follows.

1. Evidence for the relationship must be shown in a meta-analysis of either prospective cohort studies or randomised controlled trials, with an effect size significantly different to the null hypothesis (*P* < 0.05) (NB: this criterion was relaxed for smoking-disease relationships as there were no meta-analyses available).
2. The relationship must not be a comparison of “high risk” versus “low risk” groups, where the level of exposure in high and low risk groups is ill-defined.
3. The health outcome must be a NCD (e.g., we do not include the relationship between alcohol consumption and road traffic accidents).
4. The health outcome must make a substantial contribution to NCCD mortality. In practice, we used the threshold that the NCD must have resulted in greater than 500 mortalities in the UK in 2006.

One consequence of these inclusion criteria is that the PRIME does not estimate the total impact of all health outcomes attributable to each behavioural risk factor.

The PRIME uses methods that were developed for the Global Burden of Disease (GBD) project [23, 42, 43]. The model uses data on the baseline and counterfactual distributions and the relative risk linking behaviours and disease outcomes to calculate a series of population attributable fractions (PAFs) using the general formula:

(1) $$\begin{array}{c} \text{PAF}=\;\frac{\int \text{RR}\left(x\right)P\left(x\right)dx-\int \text{RR}\left(x\right)P^{\prime }\left(x\right)dx}{\int \text{RR}\left(x\right)P\left(x\right)dx}, \end{array}$$

where RR(*x*) is the relative risk of disease for risk factor level *x*, *P*(*x*) is the number of people in the population with risk factor level *x* in the baseline scenario, and *P*′(*x*) is the number of people in the population with risk factor level *x* in the counterfactual scenario [44]. The PAFs for different risk factors are combined multiplicatively in the PRIME; that is,

(2) $$\begin{array}{c} {\text{PAF}}_{\text{TOT}}=1-\overset{n}{\underset{i=1}{\prod }}\left({1-\text{PAF}}_{i}\right), \end{array}$$

where PAF_TOT_ is the final PAF for a disease after all PAFs for risk factors 1 to *n* have been combined. For each disease included in PRIME, the age and sex specific final PAFs are then applied to the annual number of deaths in the baseline situation in order to calculate the change in number of deaths in the baseline situation.

Risk factor levels are converted to discrete bins in the internal calculations conducted by the PRIME. The integral equations described in (1) above are replaced by finite sums, where the relative risks are assumed to be equal over a small range, or “bucket,” and all of the population within that bucket is assumed to have the same risk factor level.

## 3. Statistical Methods Used by the PRIME 

There are six different types of links between risk factors and health outcomes included in the PRIME that are described in this section. These are links between

1. continuous risk factor and a health outcome mediated by a single RR parameter,
2. continuous risk factor and a health outcome mediated by categorical RR parameters,
3. categorical risk factor and a health outcome mediated by categorical RR parameters,
4. energy intake, physical activity, and BMI mediated by steady state body weight equations,
5. salt and blood pressure mediated by RCT results,
6. fatty acids and cholesterol mediated by RCT results.

### 3.1. Continuous Risk Factor and a Health Outcome Mediated by a Single RR Parameter

For most of the links included in the PRIME, the risk factor is continuous and bounded at zero (e.g., amount of fruit consumed per day) and the RR used to parameterise the relationship between the risk factor and a disease outcome describes the change in risk for a unit increase in the risk factor (e.g., change in risk for each extra portion of fruit consumed) across a given range, usually the range of measures of the risk factor found in the reviewed literature. For these links, the PRIME first describes the distribution of the risk factor within the population in both the baseline and counterfactual scenarios. It does this by assuming an underlying distribution for the risk factor (usually a lognormal distribution as this distribution is bounded at zero) which is then parameterised by up to three parameters: the mean value of the risk factor in the population, the standard deviation, and, when necessary, the percentage of the population with a zero value (e.g., for alcohol consumption, the PRIME estimates the distribution of alcohol consumed in the population in g per day of alcohol, after first removing alcohol abstainers from the distribution). Using the parameterised distributions, the PRIME then divides the population into the specified buckets for the risk factor. The RR parameter is then used to estimate the RR of the health outcome in each of the buckets using the following general formula:

(3) $$\begin{array}{c} {\text{RR}}_{i}={\text{RR}}^{(x-y)/u}, \end{array}$$

where *x* is the midpoint for bucket *i*, *y* is the midpoint for the first bucket (assigned to have an RR of 1), and *u* is the unit increase reported in the literature. For example, the colorectal cancer RR for a 10 g/d increase in fibre is 0.88 [29]. The PRIME breaks fibre intake into 22 buckets, each 2 g/d wide, with 4–6 g/d fibre intake as the lowest intake bucket (used as the reference, with RR of 1), since this was the lowest intake seen in the studies included in the meta-analysis. The RR estimated for the 3rd bucket (8–10 g/d) is 0.88^(9−5)/10^ = 0.95.

Using the RR in each bucket and the total number of deaths from the disease, the death rate per 1000 is calculated for each bucket (DR_*i*_, with DR_1_ referring to the death rate in the baseline level) using the following formula where *D* is the total mortality from the disease, *p*_*i*_ is the population in bucket *i*, RR_*i*_ is the relative risk in the bucket, and *n* is the number of buckets:

(4) $$\begin{array}{c} {\text{DR}}_{1}=\frac{1000D}{p_{1}+\sum_{i=2}^{n}\left(p_{i}\cdot {\text{RR}}_{i}\right)},\\ {\text{DR}}_{i}={\text{DR}}_{1}\cdot {\text{RR}}_{i}. \end{array}$$

In the counterfactual scenario, it is assumed that the death rates in each bucket remain the same, but the population in the bucket changes. The PRIME estimates the consequent change in the number of deaths between baseline and counterfactual, which is the main outcome of the model.

The direct links between physical activity and health outcomes included in the PRIME are parameterised in a similar way, but with a slight modification. The meta-analyses used to parameterise the physical activity links suggest that the dose-response relationship is not log-linear as described previously but follows a 0.25 power transformation (these meta-analyses are currently unpublished but follow a similar transformation as described by Woodcock et al. [45]). Essentially, this transformation ensures that the effect of physical activity on health diminishes more quickly than a log-linear transformation allows. The distribution of physical activity is parameterised in the PRIME as MET hours per week in moderate or vigorous physical activity (MVPA) and requires estimates of mean and standard deviation of MET hours per week and the percentage of the population who are sedentary (i.e., having zero MET hours per week of MVPA). The RR in bucket *i* of MET hours per week is then parameterised from the RR described in the meta-analyses using the following formula, where *x* is the midpoint of bucket *i* and *u* is the unit increase in physical activity reported in the meta-analysis:

(5) $$\begin{array}{c} {\text{RR}}_{i}=1+\left(\frac{\text{RR}-1}{u^{0.25}}\right)x^{0.25}. \end{array}$$

### 3.2. Continuous Risk Factor and a Health Outcome Mediated by Categorical RR Parameters

Many of the links in the PRIME are parameterised by a set of categorical RRs which are assumed to be constant over the risk factor range that they describe. This is the case for many of the links between alcohol consumption and disease, where the J-shaped relationship cannot easily be parameterised by a single RR estimate. In these instances, the buckets used for the PRIME are set to be the same size as the categories for which the RRs are provided, and the PRIME proceeds as described above, using the RRs for each bucket that are reported in the meta-analysis.

### 3.3. Categorical Risk Factor and a Health Outcome Mediated by Categorical RR Parameters

Tobacco smoking is the only categorical risk factor that is included in the PRIME. Three categories are used: current smoker, former smoker, and never smoked. The RRs for the relationship between smoking and health outcomes are drawn from large prospective cohort studies [33–37]. The PRIME proceeds as above, but with only three buckets representing the three smoking categories.

### 3.4. Energy Intake, Physical Activity, and BMI Mediated by Steady State Body Weight Equations

The PRIME calculates the change in the distribution of BMI in the counterfactual scenario by using equations derived by Christiansen and Garby [39] to estimate the new steady state body weight that would be produced after a change in energy balance (i.e., either changes to total energy intake or total energy output). The equation used in the PRIME is given below, where BW is steady state body weight measured in kg, EI is energy intake measured in MJ per day, and PAL is physical activity level, a ratio of the total energy expenditure over resting energy expenditure. In these equations *k* is a constant term that is based on both fundamental principles of energy conservation and directly measured data and takes the value of 17.7 for men and 20.7 for women:

(6) $$\begin{array}{c} \Delta \text{BW}=k\cdot \Delta \left(\frac{\text{EI}}{\text{PAL}}\right). \end{array}$$

This change in body weight can be applied to baseline population estimates of height and BMI distribution to estimate the counterfactual BMI population distribution.

The energy intake parameter is directly entered by the user. The PAL parameter is estimated by the PRIME on the basis of the physical activity distribution described earlier. The PRIME also requires estimates of the intensity of both moderate to vigorous physical activity (MVPA) and non-MVPA within the population, which the user can define. A sensitivity analysis has shown that changes in BMI (and hence BMI-related health outcomes) in the PRIME are sensitive to the selection of the intensity parameters [46], so users should select these parameters carefully. In this sensitivity analysis, it was shown that applying a 5 METhr/week increase in physical activity to the UK population resulted in differences in average bodyweight ranging from 0.4 kg to 0.7 kg depending on the selection of the intensity of physical activity during MVPA and non-MVPA time.

### 3.5. Salt and Blood Pressure Mediated by RCT Results

The relationship between salt intake and blood pressure is mediated in PRIME by a meta-analysis of randomised controlled trials of modest salt reduction on free living individuals with duration of at least four weeks [10]. The meta-analysis estimated that a 6 g/d reduction in salt intake would result in a 5.8 mmHg reduction in systolic blood pressure. In the PRIME, this parameter is used to convert the distribution of salt consumption in the population to a distribution of “salt-related blood pressure” (e.g., those in the population who consume 4 g/d salt have salt-related blood pressure that is 5.8 mmHg less than those who consume 10 g/d). The salt-related blood pressure variable is then associated with health outcomes using the same methods as those described above.

### 3.6. Fatty Acids and Cholesterol Mediated by RCT Results

The link between fatty acid intake and blood cholesterol levels is parameterised in PRIME in a similar way to the link between salt and blood pressure. The mutually adjusted parameters linking total fat, saturated fat, monounsaturated fatty acids (MUFAs), polyunsaturated fatty acids (PUFAs), and dietary cholesterol with blood cholesterol levels are taken from a meta-analysis of controlled lab-based feeding studies [38]. In order to convert five distributions of fatty acid intake into a single distribution of “fatty acid-related blood cholesterol,” we assumed that the fatty acid intakes within a population are normally distributed. We then combined mean values of these fatty acid intakes using the following equation, where fBC is mean fatty acid-related blood cholesterol, *f*_*i*_ is mean intake of fatty acid *i*, and *α*_*i*_ is the mutually adjusted parameter drawn from the meta-analysis:

(7) $$\begin{array}{c} \text{fBC}=\overset{5}{\underset{i=1}{\sum }}\alpha_{i}{\cdot f}_{i}. \end{array}$$

To estimate a distribution of fatty acid-related blood cholesterol in both the baseline and counterfactual scenarios, it is necessary to estimate the variance by combining estimates of the variance in intake of the individual fatty acids. To do this, we use a covariance matrix derived from an analysis of the Health Survey for England 2006 [47] (NB: users are free to change the values of this covariance matrix to suit other populations) and combine the variances using the general formula for adding the variance of normally distributed variables *X* and *Y* given below, where *σ*_*X*+*Y*_ is the variance of the combined distribution, *σ*_*X*_ is the variance of distribution *X*, *σ*_*Y*_ is the variance of distribution *Y*, and *ρ* is the correlation coefficient between distributions *X* and *Y*:

(8) $$\begin{array}{c} \sigma_{X+Y}=\;\sqrt{\sigma_{X}^{2}+\sigma_{Y}^{2}+2\rho \sigma_{X}\sigma_{Y}}. \end{array}$$

## 4. Development of Source Code for the PRIME Python Program and Web Application

To improve the availability, utility, and transparency of PRIME, the model has been rewritten and converted to a custom computer program, written in Python. This program has been used to create a web application to provide an accessible and easy-to-use interface to PRIME. The code for this program has been packaged and released as an open-source project under a BSD (Berkeley Software Distribution—http://opensource.org/licenses/bsd-license.php) licence, for users to analyse, improve, and integrate into their own systems as desired. Both code and documentation are available at https://github.com/cpnp/prime.

**Figure 2:**
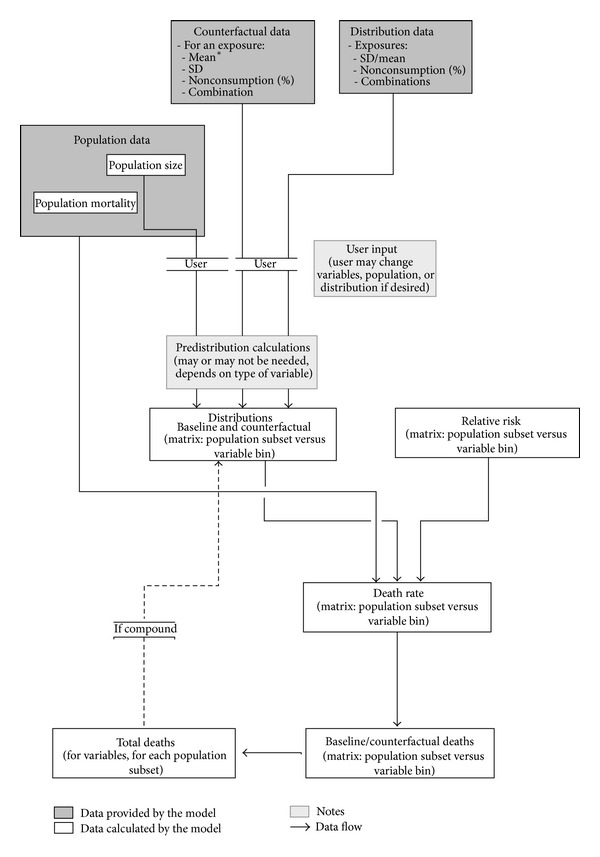
Data flow model for the open-source PRIME Python application.

The underlying data model has been restructured and rewritten, based on the original Excel model, and is now an object-oriented Python program.  Figure 2 shows an overview of the data model and data flow within the program. Although the project and code may change over time, this core structure will remain a central reference. The program has been written using Python (v 2.7.6), a powerful and flexible computer language that can be used to create different types of application, including command line, desktop, and web applications. In addition, the program uses a number of well-established 3rd party Python libraries, including numpy and scipy [48] that extend its mathematical and scientific capabilities. SQLAlchemy [49], another Python library, is an object-relational mapper, which makes it possible to use different database engines to store the underlying population and mortality data, and handles database interactions. The default database is MySQL (v 5.6.17). More information on Python code libraries can be found here: http://www.scipy.org/scipylib/citing.html.

Testing of the codebase is carried out whenever new components are added to ensure consistent and accurate results. This includes a set of tests to compare results given by the Python model to those results given for baseline data by the Excel model. A further advantage of rewriting PRIME in Python is that the time taken to run Monte Carlo analyses has been significantly reduced compared to the time taken for Excel macros to run the same calculations. The reduction is in the order of 15 minutes to ~5 seconds, depending on the exact queries and calculations being run. Full details of benchmarked calculations will be available in the Prime documentation in the GitHub repository.

**Figure 3:**
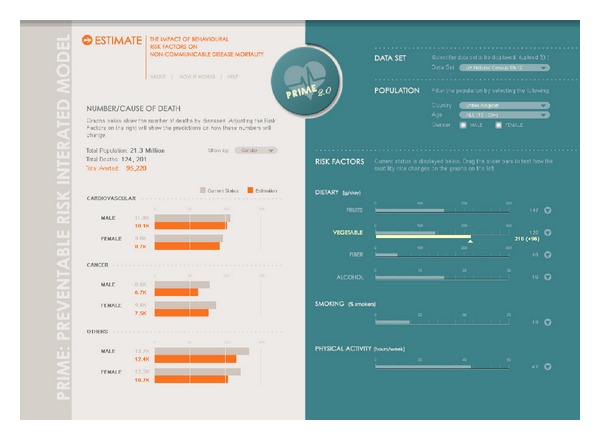
Overall layout of the main page in the PRIME web application. Input factors are set on the right, and outcomes are returned on the left.

Development of this Python package provides the ability to run the model in a number of different ways, including via a web application. The interactive web application allows users to run the model in any web browser, from anywhere with an Internet connection. It has easy controls for adjusting values of different variables, outputting resulting changes to population mortality and outcomes in an easy to use graphical interface. An overview of the design for the web application is shown in Figure 3. Two main groups of user for the web application are anticipated, who have different requirements and will access different parts of the web application according to their needs. The first of these groups is composed of users who wish to use the model to investigate the overall effect of a limited subset of variable changes. This group may comprise policy makers and public health professionals. The second group of users is those who wish to use more advanced features and perhaps investigate the inner workings of the model a little more. This group may contain other researchers who wish to use their own population datasets, alter some of the more complex variables in the model, run Monte Carlo analyses, and investigate in more detail the specifics of how the model works, for example, what assumptions are made, what distribution types are used, and which details from what publications are used in specific sections of the model. The web application offers the flexibility of providing specific features required by the user in an accessible and interactive format.

## 5. Review of Studies That Use the PRIME 

To date, eleven papers have been published in peer-reviewed journals that have used the PRIME or precursors of the model that were constructed during the development process of the model [13, 20, 22, 40, 50–56]. Earlier versions of the PRIME were sometimes referred to as DIETRON—here we refer to all the models used in these papers as PRIME. In this section, we review the results that have been produced by the PRIME. Details of the papers that are reviewed in this section are provided in Table 2, ordered chronologically.

Three of the papers have considered the health impact of achieving government public health recommendations—dietary targets in the UK [20], dietary targets in Canada [50], and safe alcohol consumption levels in the UK [13]. Of the more than 230,000 diet-related mortalities in the UK in 2007, over 33,000 (15%) could have been delayed or averted if dietary recommendations for fruit and vegetables, fibre, total fat, and saturated fat and salt were achieved. The biggest reduction in mortality would be produced by achieving the recommended five portions of fruit and vegetables per day (over 15,000 deaths averted) followed by reductions in salt consumption (over 7,500 deaths averted). These different health impacts are partly due to the distance between the dietary variable and the recommendation and partly due to the relationship between the risk factor and disease, and different results would be produced if different dietary targets were set. Since dietary recommendations are based on setting a realistic goal that individuals within the population feel is achievable and do not represent an epidemiological “minimum risk” setting [58], comparing results between dietary risk factors is somewhat arbitrary. This is demonstrated by the health impact of achieving dietary recommendations in Canada, where 36% of the 85,000 diet-related mortalities in 2004 could be averted by achieving dietary recommendations. This big increase compared to the UK figures is due to the more ambitious nature of the Canadian dietary recommendations, which recommend 8–10 portions of fruit and vegetables per day [59] compared to only 5 portions of fruit and vegetables for the UK, and 30–38 g/d fibre compared to the UK recommendation of 18 g/d. The two papers provide information to policy makers about which dietary recommendations should be prioritised in order to improve population health outcomes and both concur that the fruit and vegetables recommendation would deliver the most health benefit. However, these modelled results can only provide a partial guide for policy makers in the absence of high quality estimates of the cost-effectiveness of population-level dietary interventions [60].

The “lower risk guidelines” for alcohol consumption in England are that men should not regularly drink more than 3 to 4 units per day, and women should not regularly drink more than 2 to 3 units per day [61], where a unit is defined as 8 g of pure alcohol. The General Household Survey 2006 [62] suggested that average alcohol consumption in England was 21 g/d in men and 10 g/d in women with 20% of men and 36% of women defining themselves as nondrinkers. Therefore, moving the population to consumption levels defined by the “lower risk guidelines” would increase alcohol consumption in the population with consequently negative health outcomes. We used the PRIME to estimate the health impact of moving the mean consumption of alcohol in drinkers between 1 g/d and 48 g/d—such an analysis will balance the positive effect of alcohol on coronary heart disease [30] with the negative effects associated with some cancers [5] and liver cirrhosis [32]. The modelling suggested that the optimal level of alcohol consumption in England—that is, the level where NCD mortality was reduced to a minimum—was 5 g/d (about half a unit). Such a level of consumption would result in 4,579 (2,544 to 6,590) averted or delayed deaths a year or approximately 3% of all deaths from partially alcohol-related NCDs [13]. This total included a small increase in deaths from cardiovascular diseases that was offset by a large reduction in mortalities from cancer and liver cirrhosis.

Variants of the PRIME have been used to explore the impact of health-related food taxes and subsidies in the UK [51, 53, 54] and Ireland [52]. Two of these papers considered the potential unintended health consequences that result from substitution behaviour when certain unhealthy food items are taxed by using matrices of cross-price elasticities derived from national trends in food spending [63]. Mytton et al. [53] found that applying a 17.5% tax on food groups that are the principal source of saturated fat in the diet (whole milk, cheese, butter, cakes, pastries, and biscuits) resulted in substitutions towards salty foods and away from fruit and vegetables and as a result, despite the predicted reduction in blood cholesterol levels, the scenario resulted in an increase in mortality from cardiovascular disease. A similar scenario was investigated by Nnoaham et al. [54], also incorporating differences in results by socioeconomic groups, and the authors found that a tax on sources of saturated fat increased mortality in each income quintile in England. Both papers also considered a revenue neutral policy, where the taxes raised by targeting unhealthy foods were used to subsidise fruit and vegetables, and these analyses suggested that targeted taxes and subsidies can result in improvements of health. For example, Nnoaham et al. [54] suggested that such a scenario would result in between 3,689 and 6,435 fewer cardiovascular disease mortalities per year in England. Two papers have used the PRIME to explore the effect of taxation of sugar-sweetened beverages (SSBs) in the UK [51] and Ireland [52] on obesity levels. Both sets of analyses used an econometric model that estimated cross-price elasticities for a range of beverages to examine how different socioeconomic groups substitute between beverages after reacting to price changes. The papers suggested that a tax on SSBs (10% in Ireland and 20% in the UK) would result in a small mean reduction of total calories in the population and hence a small shift in the distribution of BMI in the population. This small shift would result in 180,000 fewer obese people in the UK. Surprisingly, the analyses suggest that this health effect would be similar in high income groups and low income groups, which suggest that the health benefit would not be progressive. This is explained by SSB consumption and SSB price sensitivity being broadly similar across income groups in both Ireland and the UK.

The PRIME has also been used to investigate the health impact of achieving low carbon diets in the UK [22, 55]. The Committee on Climate Change (CCC)—a quasi-nongovernmental organisation that advises the UK Government on progress towards meeting the 2008 Climate Change Act—included three food production scenarios in its fourth carbon budget, which were aimed at reducing greenhouse gas (GHG) emissions from the UK food sector [57]. The most dramatic of these scenarios was a 50% reduction in livestock production balanced by increases in plant commodities (since livestock, especially ruminants, has substantially higher carbon footprints than fruit, vegetables, and cereals [64]), which the CCC report estimated would result in a 19% reduction in UK agricultural GHG emissions in comparison to 2005 levels. We set out to estimate what would be the health impact of such a dramatic change in the diet. We used data on current food consumption from the Family Food Survey [65] and assumed that there would be no* intracategory* food consumption changes but that* intercategory* consumption change would follow those laid out by the CCC scenarios (e.g., in the scenario the percentage of total meat that is made up of chicken, pork, beef, and lamb would remain the same as baseline, but the total amount of meat consumption would reduce). The PRIME was used to estimate the health impact of the scenario diets and suggested that a 50% reduction in livestock would result in nearly 36,910 (30,192 to 43,592) deaths averted or delayed every year and that the main factor for this large health impact is the substitution of meat products with increased consumption of fruit and vegetables. We also investigated the impact of the scenario diet on micronutrients commonly found in animal products and found that it results in a small reduction in mean consumption of calcium and zinc, and a large reduction in vitamin B12, but an increase in consumption of iron. A second paper investigated the health impact of applying GHG emissions tax on foods in order to encourage a low GHG diet [22]. Using a method that has been proposed to estimate the shadow price of carbon [66] we set a tax level of £2.72/tCO_2_e/100 g of food and then used comparable estimates of GHG emissions for different food groups [64], combined with data on UK imports and exports to set taxation levels for different food groups. Predictably the tax was set highest for beef and lamb, with a tax level of £1.76 and £1.63 per kg of food, respectively. Using a similar econometric model as designed for the earlier SSB tax papers, we estimated the effect of this tax scenario on food consumption patterns and used PRIME to estimate the health impact. The model results suggested that a GHG tax would result in a moderately healthier diet, with 1,207 (1,003 to 1,431) deaths averted or delayed per year. The scenario also resulted in reductions of GHG emissions by over 18MtCO_2_e per year and raised annual tax revenue of over £2.0 billion.

Finally, the PRIME has also been used to estimate the proportion of geographical health inequalities in the UK that are due to variations in dietary quality [56] and to compare dietary and pharmacological approaches to prevention of heart disease [40]. The former paper applied disease-specific mortality rates from England in 2007–09 to the Scottish, Welsh, and Northern Irish populations to estimate the number of deaths that would occur in these populations if there were no inequalities in health between the four regions of the UK. The difference between actual number of deaths and the number that would occur if no inequalities existed in each country—the “mortality gap”—was then estimated. We used the PRIME to estimate the change in mortality in Scotland, Wales, and Northern Ireland in the scenario that average nutritional quality of the diet changed to equal the diet in England and used the results to estimate how much of the mortality gap could be closed if dietary inequalities were removed. The results suggested that 81% (62% to 108%) of the gap in Wales and 81% (67% to 99%) of the gap in Northern Ireland were due to differences in dietary quality, whereas only 40% (33% to 51%) of the gap between Scotland and England was due to diet. The latter paper—appearing in the Christmas edition of the BMJ—compared the effect of extending statin therapy to all over 50s in the UK with an additional portion of fruit (an apple a day) for all over 50s. The health impact of the increased statin therapy was estimated using results from a meta-analysis of RCTs [67] and the impact of the increased fruit consumption was modelled using PRIME. Both scenarios resulted in substantial decreases in annual mortality in the UK, with an estimated 9,400 (7,000 to 12,500) fewer cardiovascular mortalities in the statin scenario and 8,500 (6,200 to 10,800) fewer cardiovascular mortalities in the fruit scenario.

## 6. Future Development of the PRIME 

Development of the PRIME is currently heading towards two objectives: to share the model widely and improve transparency of methods and to develop longitudinal health outcomes. The first of these objectives is addressed by the “WEB PRIME” project, which aims to produce a user friendly web application of the PRIME and is described in the section “Development of Source Code for the PRIME Python Program and Web Application” above. We hope to launch the web application of the PRIME in 2015. One of the useful features of the PRIME is that it is reasonably straightforward to use in a number of different settings, because the data input requirements are not demanding. The requirements for the user are population-level estimates of current risk factor distributions and disease-specific mortalities. However, a drawback of this simplicity is that the health outcomes estimated by the PRIME (deaths delayed or averted) are crude and do not allow for temporal considerations of the health impact and therefore the model cannot be used to estimate the effect of risk factor scenarios on standard epidemiological measures such as life expectancy or years of life lost or on measures that incorporate morbidity such as health-related quality of life. This drawback is being addressed by using the PRIME as an input to multistate life tables model in a project that is being funded by the European Commission which will commence in October 2014. Using methods developed for the ACE Prevention projects conducted in Australia [60], we will build life tables model for the UK using projections of CVD and cancer incidence and mortality rates. The PRIME will be used to estimate PAFs (as described in “The Structure of the PRIME” above) for scenarios, and these PAFs will be applied to projected incidence rates in the multistate life tables model in order to estimate disease progression in the modelled scenario. Comparison of baseline and scenario results will allow us to estimate longitudinal health impacts of changes in NCD risk factors using the PRIME.

## 7. Future Challenges for NCD Scenario Modelling

There are a number of different NCD scenario models that have been developed with different aims and objectives in mind. Some are designed to estimate the impact of changes in risk factors on future health outcomes (e.g., the UK Health Forum CVD microsimulation model [19] or the DYNAMO model [18]). Some are designed to estimate the cost-effectiveness of screening or preventative treatments for NCDs (e.g., the CISNET life history models assessing cost-effectiveness of cancer screening [68]), and some are designed to provide a comparable framework for estimating the cost-effectiveness of NCD prevention and treatment (e.g., the ACE Prevention model [60]). It is important to be able to compare both the methods used and the results produced by these different models in order to have a greater understanding of the impact of modelling assumptions. Recently, some work has been conducted to compare the results of different CHD policy models [69] and where possible further model comparisons are needed. However, such projects are not easy, as subtly different inputs and outcomes included in scenario models mean that it is difficult to find scenarios that can be run by different models. However, lessons could be learnt from the Agricultural Model Intercomparison and Improvement Project (AGMIP), a project that has developed common future climate and population scenarios with which to compare the results of nine agricultural trade models [70]. The results of such comparisons can provide us with greater confidence of scenario results when the models agree and also provide an assessment of structural uncertainty around modelling results which is usually not addressed by individual scenario modelling analyses. Here, “structural uncertainty” refers to the uncertainty in model results that is a result of the underlying assumptions and structure of the model that generated the results and is separate from “parametric uncertainty” which refers to the uncertainty that is a result of parameters in the model being measured with error (e.g., the PRIME uses relative risks from meta-analyses as parameters, which are accompanied with error). In order to aid comparisons between models a standardised method of reporting scenario modelling results is required. Standardised reporting guidelines have been developed for subsets of NCD scenario modelling (e.g., cancer life history models [68]) but are currently not available for prevention scenarios such as those included in the analyses reviewed here.

The PRIME and most other NCD scenario models do not take account of the interaction between behavioural risk factors for NCDs. This is mainly due to the lack of data about such interaction terms within the peer-reviewed literature—there are no meta-analyses that consider the size of interactions between risk factors as they are rarely the focus of epidemiological analysis. Another reason why interaction terms are generally not considered in NCD scenario models is that it would require the models to have data on the joint distribution of risk factors within the population of interest for the baseline scenarios. In practice, such data are rarely available. If we assume that interaction terms are generally positive (i.e., that the combined risk from two risk factors is greater than the sum of its parts), then the absence of interaction terms in NCD models will result in conservative estimates of health effects. Another limitation associated with the PRIME and other cross-sectional NCD scenario models is that they are incapable of incorporating the effect of time lag between exposure and disease outcome. This produces two problems. Firstly, it is not clear when the results predicted by the PRIME could be expected to be achieved, as it is not clear how long after risk factor exposure has changed we would expect health risk to change to the levels predicted by the meta-analyses that parameterise the model. A second problem is that the PRIME does not consider lifetime exposure to risk factors when calculating PAFs. This can potentially produce quite distorting effects. For example, smoking prevalence in England peaks in early adulthood [47]. The relatively low estimate of smoking prevalence in older age groups (where the majority of NCDs occur) will underestimate the impact of smoking on NCDs as it does not adequately account for the high lifetime exposure to smoking that the older cohort has built up.

At present, there are no NCD models that are designed to predict future NCD rates which are purely based on a mechanistic relationship between risk factors and health outcomes. Longitudinal scenario models either project current trends in NCDs into the future in order to estimate baseline results (e.g., ACE Prevention [60]) or assume that NCD rates will remain constant in the baseline scenario (e.g., UK Health Forum microsimulation model [19]). This has two important consequences. The first is that NCD models are not available that can predict future changes in the course of disease, such as inflection points, that may result from increases in the prevalence of adverse risk factors such as obesity and diabetes. The lack of such models has consequences for future healthcare resource planning. The second consequence is that developed scenario models are impossible to validate against future measures of NCD rates, because scenario models are not designed to predict the future but rather to estimate the difference between two future scenarios. We do not currently have access to predictive mechanistic models because of a lack of population-level data on important epidemiological measures, such as the joint distribution of risk factors within populations, the time lag associated with risk factors and onset of disease, and interactions between different risk factors. Although joint distributions of* some* risk factors are available from national health surveys, it is rare to find a survey that measures* all* behavioural risk factors. And although single prospective cohort studies with multiple measures of risk factors can explore the time lag between exposure and health outcome, there are yet to be comprehensive meta-analyses that explore the effect of time lag. It may be possible to learn from infectious disease modellers, where model calibration is applied to estimate unknown model parameters within a known theoretical disease framework [71] in order to develop truly predictive NCD models in the future.

## 8. Conclusion

Increasing levels of computing power have allowed NCD modellers to develop more sophisticated models that can provide insight into the health impact of population-level interventions that are not well suited to standard epidemiological study designs or to extrapolate results from small studies to estimate the impact at the level of the population. The PRIME is a relatively data light model that is openly available to researchers and policy makers to estimate the population-level health impact of changes in diet, physical activity, and alcohol and tobacco consumption that will be launched as a web application in 2015. Future work to incorporate modelling developments in other fields in order to improve the PRIME and other NCD scenario models is essential in order to improve the predictive accuracy of such models and model comparison projects can allow for greater transparency, improved confidence in modelling results, and an assessment of the structural uncertainty inherent in modeling projects.

## Figures and Tables

**Table 1:** Epidemiological parameters used in the PRIME.

Link type (see “Statistical Methods Used by the PRIME”)	Risk factor	Outcome	Unit of change	Relative risk (95% confidence intervals)	Source
1	Fruit	CHD	106 g/day increase	0.93 (0.89, 0.96)	[25]
1		Stroke	106 g/day increase	0.89 (0.85, 0.93)	[26]
1		Lung cancer	80 g/day increase	0.94 (0.90, 0.97)	[5]

1	Vegetables	CHD	106 g/day increase	0.89 (0.83, 0.95)	[25]

1	Fibre	CHD	10 g/day increase	0.81 (0.72, 0.92)	[27]
1		Stroke	7 g/day increase	0.93 (0.88, 0.98)	[28]
1		Colorectal cancer	10 g/day increase	Men: 0.88 (0.78, 0.99) Women: 0.92 (0.87, 0.98)	[29]

1	Serum cholesterol	CHD	1 mmol/L decrease	Under 49: 0.44 (0.42, 0.48) 50–59: 0.58 (0.56, 0.61) 60–69: 0.72 (0.69, 0.74) 70–79: 0.82 (0.80, 0.85) Over 79: 0.85 (0.82, 0.89)	[7]
1		Stroke	1 mmol/L decrease	Under 59: 0.90 (0.84, 0.97) 60–69: 1.02 (0.97, 1.08) 70–79: 1.04 (0.99, 1.09) Over 79: 1.06 (1.00, 1.13)	[7]

1	Blood pressure	CHD	20 mmHg SBP decrease	Under 49: 0.49 (0.45, 0.53) 50–59: 0.50 (0.49, 0.52) 60–69: 0.54 (0.53, 0.55) 70–79: 0.60 (0.58, 0.61) Over 79: 0.67 (0.64, 0.70)	[6]
1		Stroke	20 mmHg SBP decrease	Under 49: 0.36 (0.32, 0.40) 50–59: 0.38 (0.35, 0.40) 60–69: 0.43 (0.41, 0.45) 70–79: 0.50 (0.48, 0.52) Over 79: 0.67 (0.63, 0.71)	[6]
1		Hypertensive disease	20 mmHg SBP decrease	0.22 (0.20, 0.25)	[6]
1		Heart failure	20 mmHg SBP decrease	0.53 (0.48, 0.59)	[6]
1		Pulmonary embolism	20 mmHg SBP decrease	0.72 (0.60, 0.87)	[6]
1		Rheumatic heart disease	20 mmHg SBP decrease	0.74 (0.61, 0.89)	[6]
1		Aortic aneurysm	20 mmHg SBP decrease	0.55 (0.49, 0.62)	[6]

1	Body mass index	CHD	5 kg/m^2^ increase	Men, BMI 15–25: 1.27 (1.16, 1.39) Women, BMI 15–25: 1.01 (0.86, 1.18) Men, BMI 25–50: 1.42 (1.35, 1.48) Women, BMI 25–50: 1.35 (1.28, 1.43)	[8]
1		Stroke	5 kg/m^2^ increase	BMI 15–25: 0.92 (0.82, 1.03) BMI 25–50: 1.39 (1.31, 1.48)	[8]
1		Heart failure	5 kg/m^2^ increase	BMI 15–25: 0.93 (0.66, 1.29) BMI 25–50: 1.86 (1.55, 2.23)	[8]
1		Diabetes	5 kg/m^2^ increase	BMI 15–25: 0.96 (0.59, 1.55) BMI 25–50: 2.16 (1.89, 2.46)	[8]
1		Hypertensive disease	5 kg/m^2^ increase	BMI 15–25: 1.17 (0.77, 1.76) BMI 25–50: 2.03 (1.75, 2.36)	[8]
1		Pancreas cancer	5 kg/m^2^ increase	1.14 (1.07, 1.22)	[5]
1		Colorectum cancer	1 kg/m^2^ increase	1.03 (1.02, 1.04)	[5]
1		Breast cancer	2 kg/m^2^ increase	Under 60: 0.94 (0.92, 0.95) Over 60: 1.03 (1.01, 1.04)	[5]
1		Endometrial cancer	5 kg/m^2^ increase	1.52 (1.35, 1.72)	[5]
1		Kidney cancer	5 kg/m^2^ increase	1.31 (1.24, 1.39)	[5]
1		Gallbladder cancer	5 kg/m^2^ increase	1.23 (1.15, 1.32)	[5]
1		Kidney disease	5 kg/m^2^ increase	BMI 15–25: 1.14 (0.74, 1.77) BMI 25–50: 1.59 (1.27, 1.99)	[8]
1		Liver disease	5 kg/m^2^ increase	BMI 15–25: 0.73 (0.54, 1.00) BMI 25–50: 1.79 (1.54, 2.08)	[8]

2	Alcohol	CHD	Categorical, baseline zero consumption	<2.5 g/d: 0.92 (0.80, 1.06) 2.5–15 g/d: 0.79 (0.73, 0.86) 15–30 g/d: 0.79 (0.71, 0.88) 30–60 g/d: 0.77 (0.72, 0.83) 60+ g/d: 0.75 (0.53, 0.89)	[30]
2		Stroke	Categorical, baseline zero consumption	<2.5 g/d: 1.00 (0.75, 1.34) 2.5–15 g/d: 0.86 (0.75, 0.99) 15–30 g/d: 1.15 (0.86, 1.54) 30–60 g/d: 1.10 (0.85, 1.45) 60+ g/d: 1.44 (0.99, 2.10)	[30]
2		Diabetes	Categorical, baseline zero consumption	<6 g/d: 0.73 (0.62, 0.86) 6–12 g/d: 0.73 (0.62, 0.86) 12–24 g/d: 0.66 (0.59, 0.75) 24–48 g/d: 0.74 (0.63, 0.88) 48+ g/d: 0.93 (0.74, 1.18)	[31]
1		M/L/P cancer	Per drink per week	1.24 (1.18, 1.30)	[5]
1		Colorectal cancer	10 g/d increase	1.09 (1.03, 1.14)	[5]
1		Breast cancer	10 g/d increase	1.10 (1.06, 1.14)	[5]
1		Liver cancer	10 g/d increase	1.10 (1.02, 1.17)	[5]
2		Liver cirrhosis	Categorical, baseline zero consumption	Women, <12 g/d: 1.90 (1.10, 3.10) Women, 12–24 g/d: 5.60 (4.50, 6.90) Women, 24–36 g/d: 7.70 (6.30, 9.50) Women, 36–48 g/d: 10.10 (7.50, 13.50) Women, 48–60 g/d: 14.70 (11.00, 19.60) Women. 60+ g/d: 22.70 (17.20, 30.10) Men, <12 g/d: 1.00 (0.60, 1.60) Men, 12–24 g/d: 1.60 (1.40, 2.00) Men, 24–36 g/d: 2.80 (2.30, 3.40) Men, 36–48 g/d: 5.60 (4.50, 7.00) Men, 48–60 g/d: 7.00 (5.80, 8.50) Men. 60+ g/d: 14.00 (11.70, 16.70)	[32]

3	Tobacco	CHD	Categorical, baseline never smoked	Men, <65, current: 2.60 (2.40, 2.90) Men, <65, former: 1.60 (1.40, 1.70) Men, 65+, current: 1.50 (1.30, 1.60) Men, 65+, former: 1.20 (1.10, 1.30) Women, <65, current: 3.20 (2.80, 3.60) Women, <65, former: 1.40 (1.20, 1.70) Women, 65+, current: 1.70 (1.60, 1.90) Women, 65+, former: 1.40 (1.30, 1.50)	[33]
3		Stroke	Categorical, baseline never smoked	Men, <65, current: 2.40 (1.80, 3.00) Men, <65, former: 1.00 (0.80, 1.40) Men, 65+, current: 1.50 (1.20, 1.80) Men, 65+, former: 1.00 (0.90, 1.20) Women, <65, current: 3.80 (3.10, 4.70) Women, <65, former: 1.50 (1.10, 2.00) Women, 65+, current: 1.60 (1.40, 1.90) Women, 65+, former: 1.20 (1.00, 1.40)	[33]
3		Diabetes	Categorical, baseline never smoked	Current: 1.44 (1.31, 1.58) Former: 1.23 (1.14, 1.33)	[34]
3		M/L/P cancer	Categorical, baseline never smoked	Current: 6.98 (3.14, 15.50) Former: 4.65 (3.35, 6.45)	[35]
3		Oesophagus cancer	Categorical, baseline never smoked	Current: 3.57 (2.63, 4.48) Former: 1.18 (0.73, 1.91)	[35]
3		Lung cancer	Categorical, baseline never smoked	Current: 8.96 (6.73, 12.10) Former: 3.85 (2.77, 5.34)	[35]
3		Pancreas cancer	Categorical, baseline never smoked	Current: 1.70 (1.51, 1.91) Former: 1.18 (1.04, 1.33)	[35]
3		Endometrium cancer	Categorical, baseline never smoked	Current: 0.74 (0.64, 0.84) Former: 0.88 (0.78, 0.99)	[36]
3		Kidney cancer	Categorical, baseline never smoked	Current: 1.52 (1.33, 1.74) Former: 1.25 (1.14, 1.37)	[35]
3		Stomach cancer	Categorical, baseline never smoked	Current: 1.64 (1.37, 1.95) Former: 1.31 (1.17, 1.46)	[35]
3		Liver cancer	Categorical, baseline never smoked	Current: 1.56 (1.29, 1.87) Former: 1.49 (1.06, 2.10)	[37]
3		Cervix cancer	Categorical, baseline never smoked	Current: 1.83 (1.51, 2.21) Former: 1.26 (1.11, 1.42)	[35]
3		Bladder cancer	Categorical, baseline never smoked	Current: 2.77 (2.17, 3.54) Former: 1.72 (1.46, 2.04)	[35]
3		COPD	Categorical, baseline never smoked	Men, current: 10.80 (8.40, 13.90) Men, former: 7.80 (6.10, 9.80) Women, current: 12.30 (9.90, 15.20) Women, former: 8.90 (7.10, 11.10)	[33]

1	Physical activity	CHD	11.25 METhr/wk increase	0.81 (0.75, 087)	Manuscript in preparation
1		Stroke	11.25 METhr/wk increase	0.79 (0.68, 0.92)	Manuscript in preparation
1		Heart failure	11.25 METhr/wk increase	0.86 (0.82, 0.89)	Manuscript in preparation
1		Breast cancer	11.25 METhr/wk increase	0.91 (0.87, 0.95)	Manuscript in preparation
1		Lung cancer	11.25 METhr/wk increase	0.74 (0.63, 0.86)	Manuscript in preparation
1		Stomach cancer	11.25 METhr/wk increase	0.74 (0.64, 0.85)	Manuscript in preparation

	Food component	Outcome	Unit of change	Regression parameter (95% confidence intervals	Source

6	Total fat	Total serum cholesterol (mmol/L)	1% of total calories increase	0.020 (0.010, 0.030)	[38]

6	Saturated fat	Total serum cholesterol (mmol/L)	1% of total calories increase	0.052 (0.046, 0.058)	[38]

6	MUFAs	Total serum cholesterol (mmol/L)	1% of total calories increase	0.005 (−0.001, 0.011)	[38]

6	PUFAs	Total serum cholesterol (mmol/L)	1% of total calories increase	−0.026 (−0.034, −0.018)	[38]

6	Dietary cholesterol	Total serum cholesterol (mmol/L)	1 mg/d increase	0.001 (0.001, 0.001)	[38]

5	Salt	Systolic blood pressure (mmHg)	6 g/day reduction	−5.80 (−2.50, −9.20)	[10]

4	Total energy intake/physical activity level	Change in body weight (kg)	1 MJ/PAL increase	Men: 17.7 Women: 20.7	[39]

*Note.* Table first appeared in [40].

**Table 2:** Published results using the PRIME and earlier versions.

Scenario	Population of interest	Changes to lifestyle parameters	Baseline health burden (annual deaths, unless specified otherwise)^1^	Primary scenario results (change in deaths per year)	Secondary scenario results	Reference
Impact of 17.5% tax on foods containing saturated fat	UK, 2003	*Total energy intake:* +2.2% *Saturated fat:* −0.13% energy (absolute) *Salt:* +5.2% *Fruit and vegetables:* −1.2%	*CVD:* 232,722	*CVD:* +2,500 to +3,500	*Tax scenario based on nutrient profile model including saturated fat, salt, and sugar, annual deaths:* −2,100 to −2,500	[53]

Impact of 32.5% tax on unhealthy foods combined with 17.5% subsidy on fruit and vegetables	UK, 2005	*Total energy intake:* +0.35% *Saturated fat:* +0.84% *Salt:* −0.45% *Fruitand vegetables:* +10.95%	*TOTAL:* 198,552 *CHD:* 100,936 *Stroke:* 57,646 *Cancer:* 39,970	*TOTAL:* −3,689 to −6,435 *CHD:* −1,584 to −1,776 *Stroke:* −1,507 to −1,532 *Cancer:* −597 to −3,127	*Percentage of weekly income lost to tax:* lowest income quintile—0.86 (0.70, 1.01) Highest income quintile—0.09 (0.07, 0.10)	[54]

Population achieves government recommendations for healthy diet	UK, 2007	*Fruit and vegetables: *347 → 440 g/d *Fibre: *15.4 → 18.0 g/d *Total fat: *37.2 → 33.0% energy *Saturated fat: *14.1 → 10.0% energy *Salt: *8.9 → 6.0 g/d	*TOTAL:* 193,947 *CHD:* 91,458 *Stroke:* 53,186 *Cancer:* 49,303	*TOTAL:* −33,157 (−29,055, −37,246) *CHD:* −20,800 (−17,845, −24,069) *Stroke:* −5,876 (−3,856, −7,364) *Cancer:* −6,481 (−4,487, −8,353)	*Percentage of dietary related deaths averted in four countries of UK:* England: 13.8% Scotland: 18.3% Wales: 15.0% Northern Ireland: 18.9%	[20]

Geographic inequality in dietary quality between Scotland and England is removed	Scotland, 2007–09	*Fruit and vegetables: *308 → 351 g/d *Fibre: *15.0 → 15.1 g/d *Total fat: *98.1 → 94.6 g/d *Saturated fat: *37.8 → 35.7 g/d *MUFA:* 36.3 → 35.3 g/d *PUFA:* 17.5 → 17.3 g/d *Dietary cholesterol:* 268 → 265 mg/d *Salt: *7.5 → 7.0 g/d	*TOTAL:* 70,753 *CHD:* 28,029 *Stroke:* 15,999 *Cancer:* 26,725	*TOTAL:* −6,353 (−5,217, −7,957) *CHD:* −3,575 (−2,896, −4,437) *Stroke:* −1,299 (−615, −2,051) *Cancer:* −1,479 (−1,170, −1,848)	*Percentage of *“*mortality gap*”* between Scotland and England closed by removing dietary inequalities:* 40% (33%, 51%)	[56]

Population achieves diet with 50% less livestock products, balanced by increase in plant commodities	UK, 2008	*Fruit and vegetables: *290 → 473 g/d *Fibre: *13.5 → 17.7 g/d *Total fat: *86.1 → 81.5 g/d *Saturated fat: *33.8 → 29.7 g/d *MUFA:* 31.5 → 29.7 g/d *PUFA:* 15.2 → 16.7 g/d *Dietary cholesterol:* 227 → 153 mg/d *Salt: *6.2 → 6.0 g/d	*TOTAL:* 191,368 *CVD:* 141,240 *Cancer:* 50,128	*TOTAL:* −36,910 (−30,236, −43,616) *CVD:* −28,674 (−22,001, −34,766) *Cancer:* −8,236 (−5,798, −10,232)	19% reduction in UK agriculture GHG emissions and 42% reduction in land use.	[55, 57]

Impact of a £2.72/tonne CO_2_e/100 g greenhouse gas tax on foods	UK, 2010	*Fruit and vegetables: *344 → 344 g/d *Fibre: *13.1 → 13.1 g/d *Total fat: *84.2 → 82.4 g/d *Saturated fat: *32.5 → 31.6 g/d *MUFA:* 31.0 → 30.3 g/d *PUFA:* 15.3 → 15.2 g/d *Dietary cholesterol:* 230 → 223 mg/d *Salt: *6.3 → 6.2 g/d	*TOTAL:* 179,615 *CVD:* 129,908 *Cancer:* 49,707	*TOTAL:* −1,207 (−1,003, −1,431) *CVD:* −961 (−723, −1,211) *Cancer:* −448 (−279, −613)	*Annual reduction in GHG emissions:* 18,683 ktCO_2_e (14,665, 22,889) *Annual revenue generated from tax:* £2,023 m (£1,980 m, £2,064 m)	[22]

Population achieving optimal level of alcohol consumption to reduce alcohol-related chronic disease	England, 2006	*Mean alcohol consumption in drinkers:* 13.1 → 5.0 g/d *Percentage of nondrinkers in population:* 29.0% → 29.0%	*TOTAL:* 170,617 *CVD:* 125,767 *Cancer:* 33,304 *Liver cirrhosis:* 5,783	*TOTAL:* −4,579 (−2,544, −6,590) *CVD:* +843 (−1,085, +2,645) *Cancer:* −2,668 (−2,103, −3,210) *Liver cirrhosis:* −2,828 (−2,508, −3,072)	*Impact of moving all nondrinkers into current alcohol consumption distribution on annual deaths:* +3,160 (−436, +6,409)	[13]

Impact of 10% tax on sugar-sweetened beverages	Ireland, 2007	*Total energy intake:* −2.1 kcal/d (−1.7, −2.6)	*Number of people with BMI* ≥ *30:* 495,300	*Number of people with BMI* ≥ *30:* −6,170 (−4,240, −8,060)	*Percentage reduction in obesity by subgroups* Low consumers: −0.5% (−0.3%, −0.6%) Regular consumers: −3.3% (−2.3%, −4.4%) Low income: −0.6% (−0.4%, −0.8%) High income: −0.7% (−0.5%, −1.0%)	[52]

Impact of 20% tax on sugar-sweetened beverages	UK, 2010	*Total energy intake:* −4.0 kcal/d (−5.2, −2.7)	*Number of people with BMI* ≥ *30:* 13,877,000	*Number of people with BMI* ≥ *30:* −180,400 (−247,100, −109,500)	*Percentage reduction in obesity by subgroups* Lowest income: −1.3% (−2.0%, −0.3%) Highest income: −2.1% (−2.9%, −1.3%) 16–29: −7.6% (−8.6%, −6.4%) 30–49: −1.3% (−1.7%, −0.8%) 50+: +0.2% (−0.2%, +0.5%)	[51]

Population achieves government recommendations for healthy diet	Canada, 2004	*Fruit and vegetables:* 460 → 963 g/d *Fibre:* 19 → 36 g/d *MUFA:* 12.7 → 14.4% energy *PUFA:* 5.5 → 6.0% energy *Saturated fat:* 10.2 → 10.0% energy *Salt:* 9.0 → 5.8 g/d	*TOTAL:* 85,527	*TOTAL:* −30,540 (−24,953, −34,989) *CVD:* −24,711 (−19,432, −28,713) *Cancer:* −5,829 (−3,985, −7,368)		[50]

Population increases fruit consumption by one portion per day	UK, 2011, adults aged 50 and over	*Fruit and vegetables:* 344 → 440 g/d (with 70% compliance)	*CVD:* 95,153	*CVD:* −8,500 (−6,200, −10,800)	*Impact of extending to population aged 30 and over, annual deaths:* −8,800 (−6,500, −11,100)	[40]

^1^Only includes deaths from diseases where risk factors that are changed in the scenario are associated with mortality (e.g., diet-related cancers include colorectal cancer, mouth, larynx and pharynx cancer, stomach cancer, and lung cancer; alcohol-related CVD includes CHD, stroke, and hypertensive disease).
